# Seasonal prevalence of hyponatremia in the emergency department: impact of age

**DOI:** 10.1186/s12873-018-0182-5

**Published:** 2018-11-15

**Authors:** Naohiko Imai, Kiyomi Osako, Nagayuki Kaneshiro, Yugo Shibagaki

**Affiliations:** Division of Nephrology and Hypertension, Department of Internal Medicine, St. Marianna University School of Medicine, 2-16-1 Sugao, Miyamae-ku, Kawasaki, Kanagawa Japan

**Keywords:** Hyponatremia, Severe hyponatremia, Emergency department, Prevalence

## Abstract

**Background:**

Hyponatremia is one of the most commonly encountered electrolyte disorders in emergency department (ED). Seasonal fluctuations of the prevalence of hyponatremia has been reported. We investigated the impact of age on the seasonal prevalence of hyponatremia in the emergency department in Japan.

**Methods:**

Total of 8377 patients presented to the ED between January 2015 and December 2016 were reviewed. The adult group aged between 18 and 64 years old consisted of 3656 patients and the elderly group aged over 65 years consisted of 4721 patients. Information collected included age, sex, serum sodium, and serum creatinine. Hyponatremia was defined as a serum sodium leve1 < 135 mEq/L and severe hyponatremia was defined as a serum sodium level < 125 mEq/L.

**Results:**

Prevalence of hyponatremia was significantly higher in the elderly group than in the adult group (17.0% vs. 5.7%, *p* < 0.001). Similarly, the prevalence of severe hyponatremia was significantly higher in the elderly group than in the adult group (1.9% vs. 0.3%, *p* < 0.001). Prevalence of hyponatremia and severe hyponatremia was significantly higher in the elderly group than in the adult group in all seasons. In the elderly group, there was a significant correlation between weather high temperature during summer and prevalence of hyponatremia (*r* = 0.510, *p* = 0.011).

**Conclusion:**

There was a major impact of age on the seasonal prevalence of hyponatremia and severe hyponatremia. Strategies to prevent hyponatremia and severe hyponatremia should be taken especially in the elderly patients during summer.

## Background

Hyponatremia is one of the most commonly encountered electrolyte disorders in the emergency departments [1, 2]. Hyponatremia is known for its high morbidity and mortality and age is a strong independent risk factor of hyponatremia [3–7]. Only a few studies have investigated the impact of age on the seasonal prevalence of hyponatremia in patients admitted to emergency departments [7, 8]. Considering the aging population in Japan, the increased susceptibility of the elderly to develop hyponatremia is particularly important. The impact of age on the seasonal fluctuations on the prevalence of hyponatremia has not been reported in Japan. Thus, we investigated the impact of age on the seasonal prevalence of hyponatremia in the emergency department in Japan.

## Methods

All adult patients (18 years old or older) who had their serum sodium levels measured at the emergency department between January 2015 and December 2016 were included. Hyponatremia was defined as serum sodium levels of less < 135 mEq/L. Severe hyponatremia was defined as serum sodium level < 125 mEq/L. Information collected included age, sex, serum sodium, and serum creatinine. We used the meteorological parameters provided by the Japan Meteorological Agency. The seasons are defined as follows: spring: March–May; summer: June–August; fall: September–November; winter: December–February. The study was approved by the Institutional Review Boards at our institution.

### Statistical analyses

Data analysis was performed using SPSS, Version 21.0 (IBM Corp, Armonk, NY). Student t test or analyses of variance were used to compare means for continuous variables. Chi-square tests were used to test statistical differences for categorical variables. A *p*-value of < 0.05 was considered statistically significant.

## Results

### Patients characteristics

From January 2015 to December 2016 a total of 8377 adult patients had their serum sodium measured at the emergency department. The key characteristics of the patients are summarized (Table 1). The mean age of the patients was 63.3 ± 22.2 years and 49.6% of patients were male. Patients were divided into adult group aged from 18 to 64 years and elderly group aged 65 years and over. The mean age of the adult group (*N* = 3656) was 41.3 ± 13.6 years and 51.5% of patients were male. The mean age of the elderly group (*N* = 4721) was 80.3 ± 8.3 years and 48.1% of patients were male.

**Table 1:** Patients characteristic

	All	Adult	Elderly
N	8377	3656	4721
Male (N)	4152	1882	2270
Age (years)	63.3 ± 22.2	41.3 ± 13.6	80.3 ± 8.3
Serum creatinine	1.03 ± 1.0	0.87 ± 0.9	1.11 ± 1.1
eGFR (mL/min/1.7m^2^)	67.5 ± 27.0	81.5 ± 23.5	56.7 ± 27.4

### Prevalence of hyponatremia and severe hyponatremia

Prevalence of hyponatremia was significantly higher in the elderly group than in the adult group (17.0% vs. 5.7%, *p* < 0.001). Similarly, the prevalence of severe hyponatremia was significantly higher in the elderly group than in the adult group (1.9% vs. 0.3%, *p* < 0.001).

### Seasonal prevalence of hyponatremia and severe hyponatremia

**Fig. 1:**
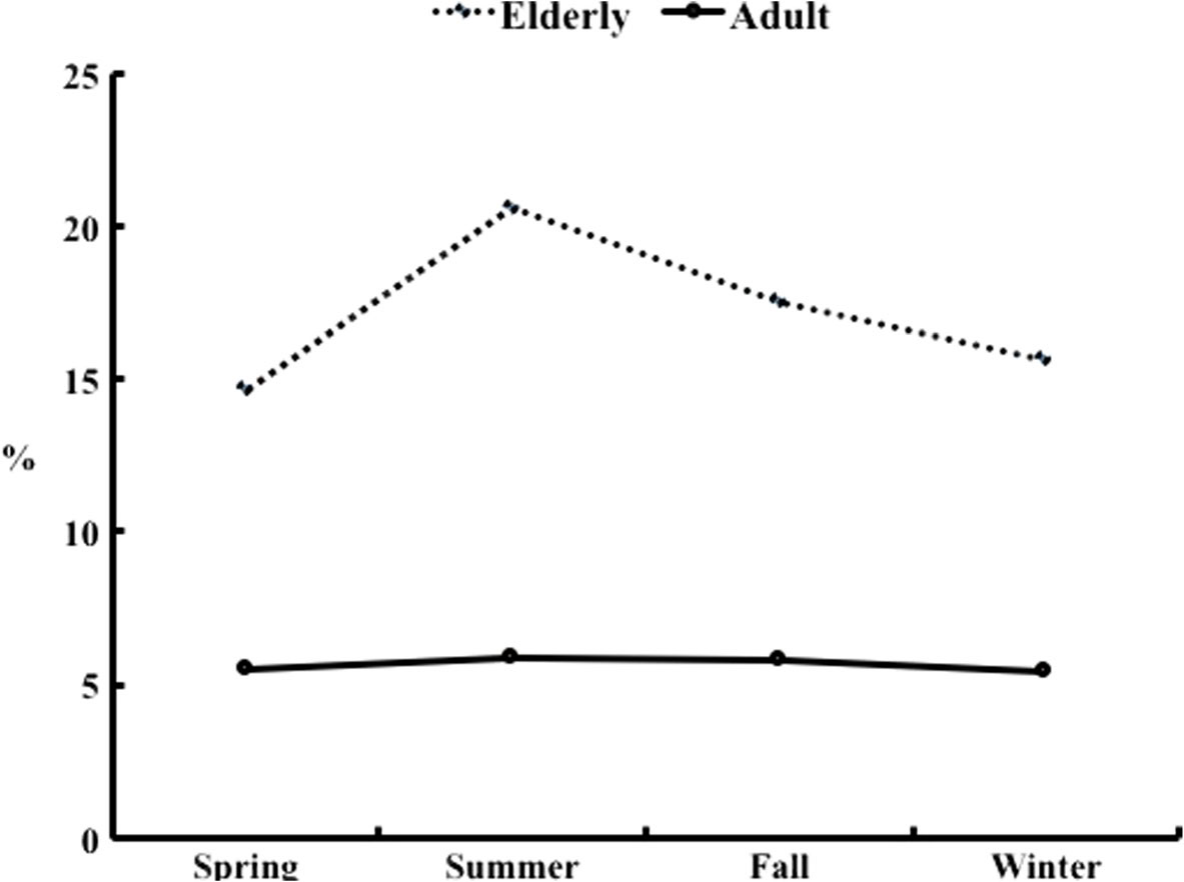
Seasonal prevalence of hyponatremia in adult group and elderly group

**Fig. 2:**
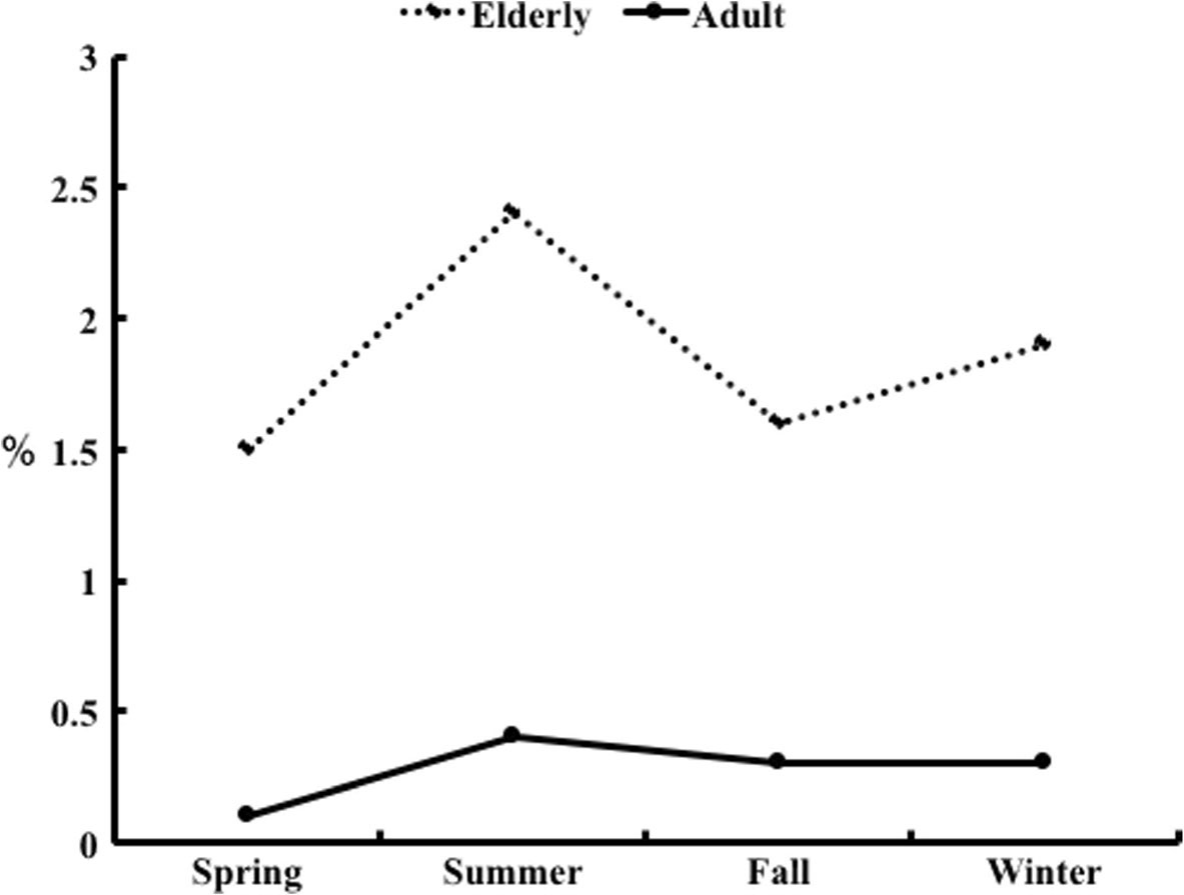
Seasonal prevalence of severe hyponatremia in adult group and elderly group

The prevalence of hyponatremia was significantly higher in the elderly group than in the adult group in all seasons (Fig. 1). Similarly, the prevalence of severe hyponatremia was significantly higher in the elderly group than in the adult group in all seasons (Fig. 2). In the adult group, the prevalence of hyponatremia and sever hyponatremia didn’t differ between the seasons (Table 2). On the other hand, in the elderly group, although the prevalence of severe hyponatremia didn’t differ between the seasons, the prevalence of hyponatremia was significantly higher in summer (*p* < 0.001 vs. spring and winter) (Table 3).

**Table 2:** Seasonal prevalence of hyponatremia and serve hyponatremia: Adult

	Spring	Summer	Fall	Winter
Hyponatremia (%)	5.5	5.9	5.8	5.4
Sever hyponatremia (%)	0.1	0.4	0.3	0.3

**Table 3:** Seasonal prevalence of hyponatremia and sever hyponatremia: Elderly

	Spring	Summer	Fall	Winter
Hyponatremia (%)	14.6	20.6*	17.2	15.6
Sever hyponatremia (%)	1.5	2.4	1.6	1.9

*p < 0.01 vs Spring and Winter

### Monthly weather temperature and prevalence of hyponatremia

**Fig. 3:**
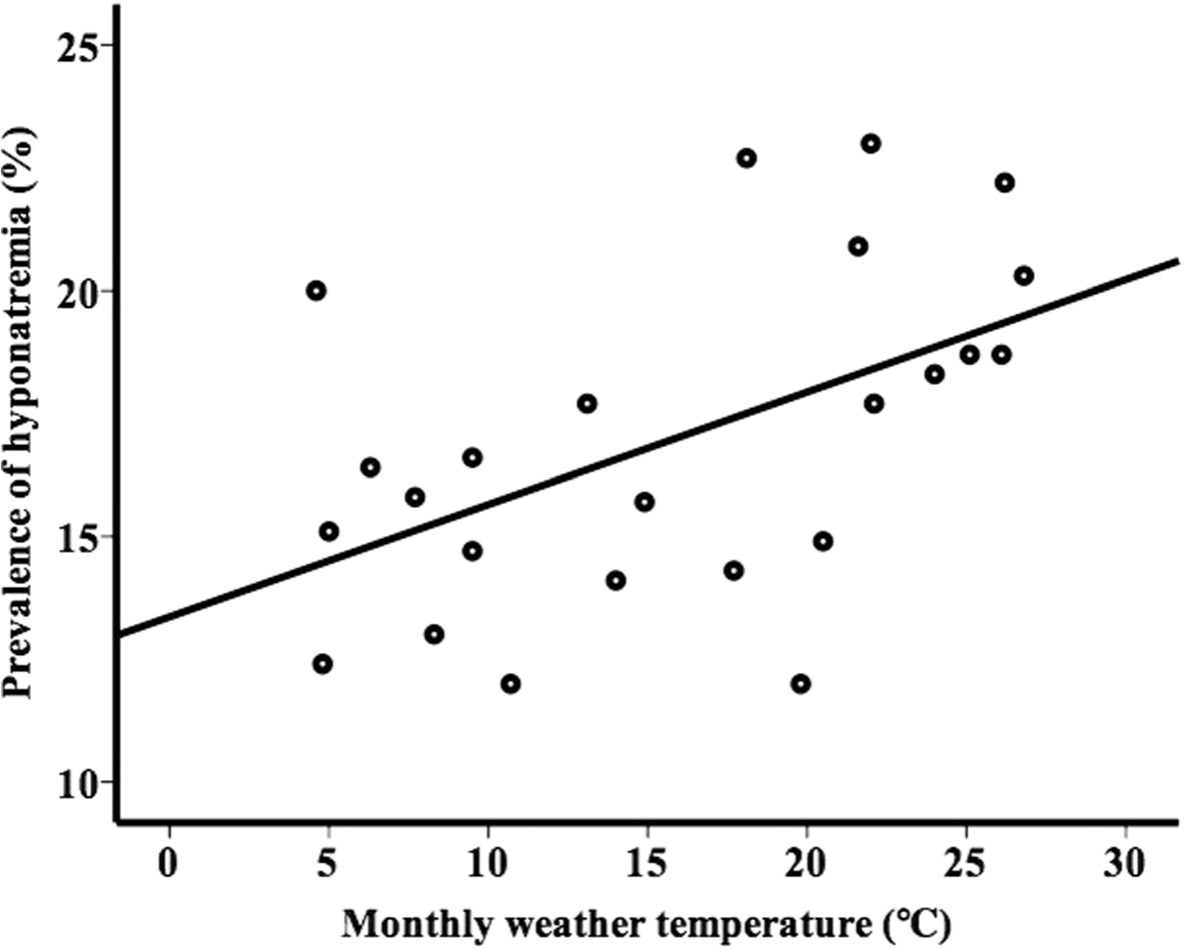
Relationship between prevalence of hyponatremia and monthly weather temperature in elderly group (*r* = 0.510, *p* = 0.011)

The monthly weather temperature during the 2-year period is summarized (Table 4). In the adult group, the monthly weather temperature didn’t show any correlation with monthly prevalence of hyponatremia (*r* = 0.094, *p* = 0.661). On the other hand, in the elderly group, the monthly weather temperature showed a linear correlation with monthly prevalence of hyponatremia (*r* = 0.510, *p* = 0.011) (Fig. 3).

**Table 4:** Mean monthly weather temperature during the 2 year period

	Jan	Feb	Mar	Apr	May	Jun	Jul	Aug	Sep	Oct	Nov	Dec
2015	4.6	5.0	9.5	14.0	20.5	21.6	26.1	26.2	22.1	17.7	13.1	8.3
2016	4.8	6.3	9.5	14.9	19.8	22.0	25.1	26.8	24	18.1	10.7	7.7

## Discussion

The prevalence of hyponatremia including severe hyponatremia was significantly higher in elderly group compared to adult group during the two-year period. Also, the seasonal prevalence of hyponatremia including severe hyponatremia was significantly higher in elderly group compared to adult group during the two-year period.

The prevalence of hyponatremia observed in emergency departments varies between studies. The 5.7% prevalence of hyponatremia in the adult group observed in our study was close to the previous report [7, 9]. Similarly, the 0.3% prevalence of severe hyponatremia in the adult group observed in our study was also close to the previous report [10]. On the other hand, the 17.0% prevalence of hyponatremia in the elderly group was higher than the previous report [7].

The prevalence of hyponatremia is strongly affected by age and is higher in elderly patients [3, 7, 8]. Various risk factors including a decline in renal function, excessive water intake, reduced salt intake, and medications could all contribute to developing hyponatremia in the elderly patients [7, 11, 12]. Although we could not assess in this study, diuretics, especially thiazides, is a known risk factors for developing hyponatremia [13, 14]. The risk associated potassium-sparing diuretics are also reported [10, 15]. It is important that physician taking care of elderly patients is fully aware of the fact that elderly patients are susceptible to develop hyponatremia because the increased morbidity and mortality associated with hyponatremia can’t be ignored [11, 15, 16].

The prevalence of hyponatremia is also strongly affected by the high weather temperature and is higher during the summer [7, 8, 10]. An increased prevalence of hyponatremia during heat periods has been reported too [11]. In our study, the prevalence of hyponatremia in the elderly group was highest during the summer. Water intake and loss are approximately 40% higher in the summer than in the winter [17]. Also, it is reported that salt appetite is not increased in summer heat [18]. These findings could have caused the high prevalence of hyponatremia during the summer in the elderly. The strong correlation between the prevalence of hyponatremia in the elderly group and the monthly weather temperature was confirmed in our study [7]. This correlation was not observed in the adult group.

This study is not without limitations. First, this is a single center study and there is a possibility of selection bias in the patients enrolled. Secondary, serum sodium was not corrected for plasma glucose levels, when elevated. Thus, patients with pseudohyponatremia are not completely ruled out. Third, several potential confounders such as reason for visit, medications which could induce hyponatremia, history of hyponatremia, and past medical history were not collected.

## Conclusions

In conclusion, we observed a major impact of age on the seasonal prevalence of hyponatremia. Elderly patients had significantly higher seasonal prevalence of hyponatremia and severe hyponatremia compared to adult patients. Strategies to prevent hyponatremia and severe hyponatremia should be taken especially in the elderly patients.

## Abbreviation

ED: Emergency department
